# The Extremophilic Actinobacteria: From Microbes to Medicine

**DOI:** 10.3390/antibiotics10060682

**Published:** 2021-06-08

**Authors:** Martha Lok-Yung Hui, Loh Teng-Hern Tan, Vengadesh Letchumanan, Ya-Wen He, Chee-Mun Fang, Kok-Gan Chan, Jodi Woan-Fei Law, Learn-Han Lee

**Affiliations:** 1 Novel Bacteria and Drug Discovery Research Group (NBDD), Microbiome and Bioresource Research Strength (MBRS), Jeffrey Cheah School of Medicine and Health Sciences, Monash University Malaysia, Bandar Sunway 47500, Malaysia; lyhui1403@yahoo.com.hk (M.L.-Y.H.); loh.teng.hern@monash.edu (L.T.-H.T.); vengadesh.letchumanan1@monash.edu (V.L.); 2 Clinical School Johor Bahru, Jeffrey Cheah School of Medicine and Health Sciences, Monash University Malaysia, Johor Bahru 80100, Malaysia; 3 State Key Laboratory of Microbial Metabolism, Joint International Research Laboratory of Metabolic and Developmental Sciences, School of Life Sciences and Biotechnology, Shanghai Jiao Tong University, Shanghai 200030, China; yawenhe@sjtu.edu.cn; 4 Division of Biomedical Sciences, School of Pharmacy, University of Nottingham Malaysia, Semenyih, Selangor 43500, Malaysia; CheeMun.Fang@nottingham.edu.my; 5 Division of Genetics and Molecular Biology, Institute of Biological Sciences, Faculty of Science, University of Malaya, Kuala Lumpur 50603, Malaysia; 6 International Genome Centre, Jiangsu University, Zhenjiang 212013, China; 7 Faculty of Applied Sciences, UCSI University, Kuala Lumpur 50600, Malaysia

**Keywords:** extremophile, actinobacteria, environment, bioactivity, metabolites

## Abstract

Actinobacteria constitute prolific sources of novel and vital bioactive metabolites for pharmaceutical utilization. In recent years, research has focused on exploring actinobacteria that thrive in extreme conditions to unearth their beneficial bioactive compounds for natural product drug discovery. Natural products have a significant role in resolving public health issues such as antibiotic resistance and cancer. The breakthrough of new technologies has overcome the difficulties in sampling and culturing extremophiles, leading to the outpouring of more studies on actinobacteria from extreme environments. This review focuses on the diversity and bioactive potentials/medically relevant biomolecules of extremophilic actinobacteria found from various unique and extreme niches. Actinobacteria possess an excellent capability to produce various enzymes and secondary metabolites to combat harsh conditions. In particular, a few strains have displayed substantial antibacterial activity against methicillin-resistant *Staphylococcus aureus* (MRSA), shedding light on the development of MRSA-sensitive antibiotics. Several strains exhibited other prominent bioactivities such as antifungal, anti-HIV, anticancer, and anti-inflammation. By providing an overview of the recently found extremophilic actinobacteria and their important metabolites, we hope to enhance the understanding of their potential for the medical world.

## 1. Introduction

Actinobacteria is a phylum of bacteria that comprises Gram-positive genera with high guanine and cytosine (G + C) content in their genomes and a few Gram-negative species [1,2]. Although actinobacteria are commonly present in terrestrial and aquatic ecosystems, they have a wide range of habitats, including extreme geographical locations such as deserts, hot springs, salt lakes, caves, and deep-sea [3,4,5,6]. In light of their abundance in such extreme environments accompanied by their well-known biosynthetic capabilities, scientists are interested in their metabolic versatility, discovering novel bioactive secondary metabolites, and their extracellular enzymes, which can be potentially propitious for pharmaceutical development [7,8,9,10,11].

Furthermore, actinobacteria have astonishing capabilities in adapting contaminated soil and efficiently decomposing organic materials such as hemicellulose and lignin through the actions of their metabolites [12]. Apparently, these bacteria can be bioindicators to toxic contaminants due to their higher sensitivity in detecting toxic elements [13]. Their unique tolerance to these contaminants accompanied by their degradation, biostimulation, and bioaugmentation abilities have enabled them to be great candidates for the bioremediation of heavy metals and organic pollutants [14,15,16]. Additionally, actinobacteria are incredible producers of agro-active and plant growth-promoting (PGP) compounds such as siderophores and indole acetic acid [17,18]. Actinobacteria have a major contribution to the agriculture industry whereby numerous strains (either single strains or in consortia) or their associated compounds have been applied as biofertilizers, biopesticides, and biological control agents [19,20,21,22]. The utilization of actinobacteria to manage plant diseases and pests that damage agricultural crops is effective, cost-saving, and eco-friendly, thus, they can substitute and mitigate the use of harmful chemical fertilizers and pesticides.

Actinobacteria can be categorized into two genera, *Streptomyces* and non-*Streptomyces* [23]. *Streptomyces* is the largest genus of Actinobacteria, and these bacteria are predominantly found in soil, but can also be present in various habitats such as marine/mangrove environments and plants [24,25,26,27]. Streptomycetes are unique filamentous Gram-positive bacteria that produce vegetative hyphae [28,29,30]. About 80% of clinically used antibiotics are derived from actinobacteria, in which a good number of them are isolated from the genus *Streptomyces* [19,31,32,33]. Astoundingly, streptomycetes remain as inexhaustible sources of antimicrobials to date [34,35]. In addition, about 17% of known active secondary metabolites are produced by this genus and many of them are of great medical significance [36,37,38,39]. For instance, Ivermectin, an antiparasitic agent is derived from *Streptomyces avermitilis* for treating lymphatic filariasis, was found to have a potent inhibitory effect on the growth of the formidable coronavirus disease 2019 (COVID-19) causative virus (SARS-CoV-2) [40,41]. Besides, streptomycetes are also recognized as producers of antifungal, antitumor/anticancer, antioxidant, and antiviral agents [42,43,44].

In recent decades, the non-*Streptomyces* which is known as the rare actinobacteria (e.g., *Micromonospora*, *Microbacterium*, *Jishengella*, *Salinispora*, *Saccharopolyspora*, *Sinomonas*, *Nocardiopsis*, etc. [45,46,47,48,49]), has piqued the scientists’ interest in discovering new unprecedented bioactive compounds produced by them. On the premise that extremophilic actinobacteria are a promising potential source of new drugs, we attempt to provide an overview of their bioprospecting aspects in this review.

## 2. Types of Extremophiles

Extremophiles are organisms that live in extreme habitats. They often have unique survival mechanisms to withstand harsh conditions such as high temperature, extreme pH, salinity, pressure, and aridity [50,51]. Extremophiles can be divided into two broad categories, namely, the extremotolerant and the extremophilic. In some cases, the scientific community applied the term “extremophilic organism” to exclusively define organisms requiring one or more extreme growth conditions. In comparison, extremotolerant organisms are those that are able to tolerate one or more physicochemical parameters [52]. Extremophile—the suffix ‘-phile’ originated from the Greek word ‘philos’, which conveys the meaning of ‘love’ and ‘preference’ of extreme environments [11,53]. Some examples of different types of extremophiles are listed in the following [54,55]: (a) thermophile—an organism that grows best at high temperatures and is commonly found in hot places such as the desert; (b) psychrophile—an organism that grows best at low temperatures; (c) halophile—an organism that thrives in habitats with high salt concentrations, such as sea and salt lakes; (d) alkaliphile—an organism that grows best in an alkaline environment; (e) acidophile—an organism that grows best in an acidic environment; (f) barophile—an organism that thrives at high-pressure conditions and is commonly found in deep-sea habitats; and (g) xerophile—an organism that grows best in an extremely arid area such as the desert. This review aims to collect information on actinobacteria present in various extreme environments and their potential to produce metabolites with bioactive properties such as antibacterial, antifungal, anticancer, and many more.

## 3. Actinobacteria in Extreme Environments

### 3.1. Extremophilic Actinobacteria in Hot Springs

Hot springs are usually formed by magma that heats the rainwater or underground water geothermally near the active volcanoes [56,57]. They are usually of low salinity (<0.5%) and have a wide range of pH values ranging from 0.5 to 9 [58]. Hot springs are for balneotherapy or recreational purposes and are a breeding ground for extremophilic actinobacteria. A thermophile is a type of extremophile that survives growth optimally at a temperature of more than 50 °C [59]. To prevent the protein from aggregating at high temperatures, they have special ‘heat shock’ proteins called chaperones responsible for unfolding the denatured protein damaged by heat [60].

In a study by Liu and colleagues [61], sediments were taken from Tengchong County of Yunnan Province in China. Fifty-eight actinobacteria isolates were recovered from 10 hot springs distributed among Hehua, Rehai, and Ruidian, in which two novel genera, *Thermoactinospora* and *Thermocatellispora*, were also identified. The sampling sites’ temperature and pH ranged from 62 °C to 99 °C, and 2.5 to 9.0, respectively. It has been reported in another study that most of the thermophilic actinobacteria found in the Rehai were able to synthesize thermostable polymer-degrading enzymes which allow the bacteria to withstand the protein-denaturing temperature [62]. In particular, one of the strains produced cellulase, β-1,4-endoglucanase (Cel5A), which was highly tolerant to a high concentration of salt [62], and thus, indicates that the bacteria could be polyextremophilic and have a halotolerant property. Surprisingly, Liu, et al. [61] found that 53/58 strains were affiliated to 12 genera, namely, *Actinomadura*, *Micromonospora*, *Microbispora*, *Micrococcus*, *Nocardiopsis*, *Nonomuraea*, *Promicromonospora*, *Pseudonocardia*, *Streptomyces*, *Thermoactinospora*, *Thermocatellispora*, and *Verrucosispora*, in which several isolates exhibited antibacterial activities against various common pathogens including *Acinetobacter baumannii*, *Micrococcus luteus*, and *Staphylococcus aureus*. Furthermore, one strain, *Micromonospora* YIM 78104, demonstrated a particular broad antibacterial property. This study suggested a variety of actinobacterial species that are yet to be explored from hot springs, in which their secondary metabolites may contribute to the development of new antibiotics.

Gholami, et al. [56] isolated a novel strain, *Kocuria rosea* MG2, from the Ab-e-Siah spring in Ramsar City in Iran with the highest natural radioactivity. Ab-e-Siah spring is a radioactive hypothermal spring with a recorded radon concentration of 146.5 Bq. l-1 [63]. Its temperature ranged from 28 to 35 °C with a pH value of 6.8 [56]. In this study, the MG2 strain was identified as *Kocuria rosea* by 16S rRNA gene sequencing. Multiple stress tests were carried out, and the results were captivating. It was found that this strain was polyextremophile and able to survive under multiple stresses such as high levels of UV-C radiation, hydrogen peroxide, and desiccation. It also exhibited maximal growth at pH 9.2. It was suggested that carotenoids played an essential role in the photoprotective mechanism of the bacteria [56]. Carotenoids can absorb maxima at 450 nm, which makes them an effective antioxidant [64]. This study provides a basis for advanced research on developing antioxidant agents with natural biomolecules.

Contrary to the traditional views and perceptions, the studies above have proven that in extremophile actinobacteria from hot springs, it is not necessary to be thermophilic. They may even be polyextremophilic, in which further investigation is required. Actinobacteria from hot springs can have various mechanisms to combat the harsh conditions of hot springs. The biometabolites they synthesized could be a potential new source of medicine such as antibiotics and antioxidants.

### 3.2. Extremophilic Actinobacteria in Deserts

Deserts cover about one-fifth of the Earth’s surface [65]. Deserts are incredibly arid (average annual rainfall less than 25 cm) [66] and have a wide range of temperatures and weather conditions with low nutrient status, making it difficult for most organisms to survive [67]. Although the general impression of a desert is patches of hot and empty land, some deserts are cold all year round. They can be classified into four categories: subtropical, cold, coastal, and semiarid [68]. Though deserts were once thought to be lifeless due to their extreme environments, recent studies have proven this perception is wrong. A wide range of actinobacteria are cultivatable in these places. To survive in such a harsh environment, living forms, including bacteria, need to have unique survival mechanisms to adapt to the extreme environment. Therefore, they tend to produce various interesting secondary metabolites which assist them in their survival.

Many studies have been carried out to investigate the actinobacteria isolated from deserts and analyze their bioactive potentials. Abenquines are new bioactive metabolites that Schulz, et al. [69] discovered. *Streptomyces* sp. strain DB634 was isolated from the soil taken from Salar de Tara of the Atacama Desert, Chile, which is known to be one of the driest places on earth with an average annual rainfall of about 15 mm; every one square meter only receives a depth of 15 mm water each year [70]. It also has the highest level of ultraviolet radiation on earth [71]. For the above reasons, its soil has been compared to that of Mars. In the study, four abenquines (A–D) were then isolated from the fermentation broth of *Streptomyces* sp. strain DB634 and found to be structurally related to aminobenzoquinones. Other studies have revealed that benzoquinones possess antioxidant and anticancer properties in addition to anti-inflammatory effects [72]. Abenquines A and D demonstrated selective inhibition of phosphodiesterase type 4b (PDE4B), which is known to upregulate CYLD expression, a key modulator in suppressing inflammatory reactions [69,73]. Hence, these two abenquines can be a potential source for developing a new anti-inflammatory agent for inflammatory diseases. Besides, inhibition of PDE4 downregulates the production of cyclic adenosine monophosphate (cAMP), which is the cardinal regulator of both the innate and adaptive immune response, and it is also capable of suppressing T-cell stimulating cytokines [74,75]. Therefore, abenquines could be an alternative therapeutic option for T-cell mediated autoimmune disorders such as celiac disease and rheumatoid arthritis, although more studies are warranted to understand its pharmaceutical applications.

Four types of ansamycin-type polyketides, the chaxamycins A–D (Table 1), were identified from the *Streptomyces* sp. strain C34 isolated from Salar de Tara of Atacama soil [76]. Ansamycin is a lipophilic antibiotic that possesses antitumor activity [76]. It exerts its activity by selectively inhibiting the heat shock protein (Hsp90) by interrupting its ATPase activity, which induces tumor cell death [76]. Its selective action against Hsp90 also leads to the degradation of proteins essential for cancer cells’ survival [77]. Moreover, the increase of antibiotic-resistant bacteria has driven scientists towards the research for new antibiotics. Thus, the antimicrobial properties of chaxamycins A–D were evaluated with *Staphylococcus aureus* and *Escherichia coli* by the agar diffusion method. Chaxamycin D exhibited a selectively high antibacterial activity against methicillin-resistant *S. aureus* (MRSA) and methicillin-sensitive *S. aureus* (MSSA), the majority with MIC values of less than 1.21 µg/mL [76]. This study exemplifies that actinobacteria isolated from deserts are promising bioprospecting resources for new antibiotics and cancer drugs.

Habitats with extreme aridity such as the Atacama Desert have drawn microbiologists’ interest due to the variety of flora found there. The compounds synthesized by these florae are viewed as the scaffolds for new drugs. Wichner, et al. [78] discovered six novel glycosides—lentzeosides A–F from the Atacama Desert (Table 1), which demonstrated anti-HIV integrase activity. The soil samples were collected from a high-altitude location (>5000 m) where *Lentzea* sp. H45 was isolated. The compounds lentzeosides A–F produced by the strain were then tested for inhibitory activity against HIV-1 integrase at different concentrations [78]. HIV-1 integrase is a vital enzyme for completing the HIV viral replication cycle at the post-entry phase and, therefore, has been the target for antiretroviral drug development [69]. The three FDA-approved antiretroviral drugs, elvitegravir, raltegravir, and dolutegravir are all integrase strand transfer inhibitors [79]. Results showed that lentzeoside C, D, and E achieved IC_50_ values at 21, 16, and 21 μM, respectively, which were lower than lentzeoside A, B, and F. This indicates that lentzeoside C, D, and E exhibit a more potent inhibitory activity, whereas lentzeoside A, B, and F demonstrate a moderate inhibitory effect on HIV integrase [78]. Hence, this newly discovered group of lentzeoside is a good product for antiretroviral therapy.

Nithya, et al. [80] evaluated the antimicrobial activity of 134 actinobacterial isolates collected from the Saudi Arabian desert. Among these isolates, the ethyl acetate extract of *Streptomyces* sp. DA3-7 demonstrated a broad-spectrum antagonistic effect on various pathogens, including *Pseudomonas aeruginosa*, *Staphylococcus aureus*, *Klebsiella pneumoniae*, *Enterococcus faecalis*, *Escherichia coli*, *Proteus vulgaris*, and *Salmonella typhimurium*; as well as Fungi *Candida albicans*, *Cryptococcus neoformans*, and *Saccharomyces cerevisiae*. It is likely that *Streptomyces* sp. DA3-7 could be a thermotolerant bacterium as it was able to tolerate maxima at 40 °C. It has been established that thermotolerant microbes achieve optimal growth at 40 °C [81,82]. Furthermore, the extract also displayed cytotoxic activity against the MCF-7 breast adenocarcinoma cell line (IC_50_ = 85 μg/mL). The active compound, pyridine-2,5-diacetamide, was isolated from the crude extract, and it was found that MIC values of pyridine-2,5-diacetamide were the lowest against *E. coli* and *C. neoformans* (both 31.25 μg/mL) (Table 1), which is lower than that of the standard therapeutic drugs ketoconazole (50 μg/mL) and streptomycin (10 μg/disc), respectively [80].

### 3.3. Extremophilic Actinobacteria in Deep-Sea Sediment

The marine creatures commonly known to us are coastal ocean species, and most of the live forms in the deep sea remain enigmatic to humans. Most of this vast blue realm is unexplored, and only a small fraction of the ocean has been mapped [83]. The pressure, oxygen level, temperature, and nutrients of the deep-sea vary depending on the area. Nevertheless, in such an extreme environment where the pressure is immense, and no light can penetrate, certain groups of actinobacteria have acclimatized and demonstrated great biosynthetic capacity [84,85]. Research on deep-sea actinobacterial diversity is limited, which is often due to the difficulty of sampling. Thanks to the breakthrough of technologies, more unique species are brought to light.

Eighteen marine actinobacteria were isolated from seawater, corals, and echinoderms in Avilés Canyon, Spain. Samples were collected at a depth of 1500 to 4700 m. As determined by 16S rRNA sequencing, they mostly belonged to the genus *Streptomyces*, and the remaining were *Pseudonocardia*, *Micromonospora*, and *Myceligenerans* [86]. Cytotoxic assays of ethyl acetate extract of the strains against HeLa, a breast cell line, and HCT116, a human colon tumor cell line, were also carried out. The extracts of the two strains *Streptomyces cyaneofuscatus* M-157 and M-192 showed the highest cytotoxic activity against the cancer cells. Even more so, both extracts were still active even after dilution at 1:100. Both *Streptomyces xiamenensis* M186 and *S. cyaneofuscatus* M190 were also able to produce β-elemene, a compound that has been used to treat brain and breast cancer clinically [87,88]. Metabolite profiling analysis showed that three compounds, cosmomycin, daunomycin, and galtamycin which possess antitumor activity, were detected in the ethyl acetate extract of *S. cyaneofuscatus* M192 [86]. Besides, antibiotic assays with ethyl acetate extracts from different strains also showed potent antibacterial activity against a wide range of pathogens and fungi such as Gram-negative *Escherichia coli, Micrococcus luteus*, and *Saccharomyces cerevisiae*. Two of the strains, *Micromonospora tulbaghiae* M194 and *Streptomyces halstedii* M204, showed a moderate antifungal effect on *S. cerevisiae* [86]. Notably, only the strains of *S. cyaneofuscatus* produce compounds with antagonistic activity against antibiotic-resistant *M. tuberculosis*. Based on these findings, these strains’ extract exerted good antimicrobial activity towards several pathogens and cytotoxic effect against HeLa and HCT116 cancer cells (Table 2). In short, this study presented a preliminary finding that marine actinobacteria can be a great potential source of antifungal and anticancer agents other than antibacterial. Therefore, it will be worthy of investigating the compounds responsible for these observed bioactivities.

Unarguably, the active metabolites of actinobacteria are indeed a great source of new drugs. Nevertheless, the application of elicitor to the culture medium is crucial to stimulate the stress response for metabolites’ production. Factors such as type, concentration, and duration of exposure of the elicitor are also cardinal to determine the production of the metabolites [89]. Xu, et al. [90] evaluated fifty actinobacteria strains cultivated from deep-sea at various sites where the depths ranged from 150 fsw to 2790 fsw. Nineteen of the isolates belonged to the genus *Streptomyces* while the others were rare actinobacteria. Notably, 27 strains showed positive antimicrobial activity, whereby the activity of 15 strains was enhanced by the elicitor lanthanum chloride (LaCl_3_) (2 mM) while 11 of them were attenuated by it. For instance, *Streptomyces* sp. R818 exerted a potent antifungal (MIC of 25 µg/mL) and synthesized antimycin-like metabolite, urauchimycin D, only when LaCl_3_ was used as the elicitor. Until now, metronidazole or vancomycin remains the first-line therapy for *C. difficile* infection [91]. Interestingly, the antibacterial activity against *C. difficile* was detected in *Salinispora* M864 after the fermentation with LaCl_3_. It exerted its activity with an MIC value (0.125 µg/mL) four times less than that of metronidazole and vancomycin (0.5 µg/mL) [90]. Herein, a pertinent elicitor is essential for obtaining the desired bioactivity effectively. The significant bioactivities of the strains are summarized in Table 2.

### 3.4. Extremophilic Actinobacteria in Caves

There is an abounding number of caves on earth, and the most common types are the limestone, calcareous, and basaltic caves [92]. Depending on the types of caves, the processes of formation range widely. For instance, stone caves are formed by erosion and weathering over millions of years [93], while limestone caves are formed by natural acid dissolving the stone [94]. Some caves, such as moonmilk caves, are formed by microbial degradation of carbonate [92].

Moonmilk has long been regarded as a medication. From the 16th to 19th centuries, moonmilk was used as a medication to treat calcinosis and cardialgia, according to swiss naturalist Conrad Gesner (1516–1555) [95]. Though not all moonmilk caves’ formation involves microbial activity, many do hold a wide range of microbes such as bacteria, especially streptomycetes, fungi, and algae in markedly high density [92,96]. Forty isolates were obtained by Adam, et al. [97] from the moonmilk cave Grotte des Collemboles, Comblain-au-Pont located in Belgium. These isolates were associated with the genera *Agromyces, Amycolatopsis, Kocuria, Micrococcus, Micromonospora, Nocardia, Rhodococcus*, and *Streptomyces*. The extremophiles have to develop unique survival strategies that allow them to dwell in the moonmilk cave exclusively [92]. This characteristic is evidenced by the highly territory-selective behavior of the isolates. For instance, 58% of the isolates in pure cultures died after the second round of inoculation in the study. It is likely to be caused by the absence of neighboring cultures and the substances emanated by them, a common mutualistic survival strategy adopted by organisms dwelling in an oligotrophic environment [97]. The antibacterial activity of the isolates was evaluated via the cross-streak method. Overall, the isolates showed a more potent inhibitory activity against Gram-positive bacteria than Gram-negative bacteria. Among all isolates, one extremely rare actinobacterium *Amycolatopsis* sp. MMun171 (actinobacterial abundancy <0.001%) exhibited the most robust antibacterial activities against both Gram-positive and Gram-negative microbes (*E. coli, P. aeruginosa, Citrobacter freundii, K. pneumoniae, Bacillus subtilis, S. aureus, and M. luteus*) under all culture conditions [97] (Table 3). This finding rekindles the hope of researchers to search for novel antibiotics from extremophiles in unique niches. However, since many isolates (58%) were lost during the purification process in the first study, it is necessary to mimic their environmental niche with specific growth factors to increase microbial growth.

A wide diversity of taxonomy, including some rare taxa, were isolated from the Shuanghe Karst Cave in Guizhou province in China. It is the longest cave in Asia, with a total cave passage of 130 km [98]. Karst caves are formed by the slow dissolution of limestone, gypsum, and dolomite by acid rainwater [99]. The cave is an extreme habitat because it is dim, humid, and cold but is also oligotrophic as a minimal source of organic material is present. A total of 45 isolates categorized into 23 species and 7 genera in which most of them were *Streptomyces* (52%), followed by *Actinoplanes* (13%), *Nocardioides*, *Agromyces*, *Rhodococcus*, *Oerskovia*, and *Micromonospora* (all >1%) were investigated by Long, et al. [100]. The antimicrobial activity of these isolates was screened, and 16 out of 45 isolates showed inhibitory activity against at least one of the tested pathogens *E. coli*, *S aureus*, and *Botrytis cinerea*. Besides, *Streptomyces badius* S142 and *Actinoplanes friuliensis* S761 displayed the strongest activity against all pathogens. This result is in line with a previous study in which an amphomycin-like new lipopeptide compound, friulimicins, derived from *A. friuliensis* demonstrated an intense antibiotic activity, even against multidrug-resistant strains [101]. In short, these studies have exemplified the high diversity of rare actinobacteria in caves, and the bioactive compounds produced by these extremophiles in these particular niches do offer a promising means to tackle the antibiotic resistance crisis.

### 3.5. Extremophilic Actinobacteria in Salt Lakes

Salt Lakes are one of the unique niches that have drawn the interest of scientists in recent years. A salt flat is the basis of the formation of salt lakes. The salt flat is usually formed in arid areas where evaporation outpaces precipitation, leaving the salt behind [102]. When there is open water such as rain and stream entering the landscape that dissolves the salt precipitate, a salt lake is formed [102]. Interestingly, they display a broad diversity in their sedimentary process, morphology, hydrology, and ecosystems [103]. Depending on the content of the lakes, their composition of ecosystems varies greatly. Given their high saturation of ions, microbial dwellers often develop unique strategies to cope with extreme conditions.

Generally, the resistance mechanism and DNA-repair system of extremophilic bacteria in the salt lake were investigated by Albarracín, et al. [104]. A group of bacteria belonging to the *Acinetobacter* genus was obtained from the high-altitude Andean lakes (HAAL), Puna Desert. HAALs is a collection of salt lakes located at the Dry Central Andes where UV-B radiation is exceptionally high. It is also characterized by high arsenic toxicity, salinity, extreme temperatures, and pH [105]. The potent photo-repair ability of the extremophiles might be due to a particular gene *HQ443199 of Ver 3*, which encodes class-I photolyase responsible for repairing UV-induced DNA lesions in *cis*-syn cyclobutane pyrimidine dimer (CDP) and pyrimidine (6-4) pyrimidone photoproducts (6-4PPs), which are commonly damaged by high UV-intensity [106]. A study by Wu et al. [107] revealed a rich diversity of actinobacteria (*Actinomycetes*, *Bifidobacterium*, *Corynebacterium*, etc.) present in the sediments of two salt lakes, Qaidam Lake and Qinghai Lake, China. Nonetheless, the coping mechanism of these extremophilic actinobacteria requires further investigation.

In extreme environments where nutrients and resources are scarce, organisms tend to produce antimicrobial secondary metabolites to inhibit other competitors’ growth for survival. Therefore, niches such as HAALs are storehouses of potential sources of antibiotics. The extremophilic profile and antimicrobial activity of actinobacteria in HAAL are investigated in a study conducted by Rasuk, et al. [105]. Fifty-one isolates were from various lakes of HAALs and were found to be members of the following genera: *Arthrobacter*, *Blastococcus*, *Brevibacterium*, *Citrococcus*, *Kocuria*, *Microbacterium*, *Micrococcus*, *Micromonospora*, *Nesterenkonia*, *Rhodococcus*, as well as *Streptomyces*. Their polyextremophilic properties were evaluated. Results showed that all 51 isolates demonstrated high resistance to UVB radiation. Furthermore, several isolates were able to tolerate and grow in an extremely high pH value of 12, indicating that they are incredibly alkaliphilic. Regarding their halotolerant property, all strains could tolerate 5% NaCl, but only 21 of the isolates were able to tolerate up to 25% NaCl. The antagonistic activities were studied against *S. aureus, E. coli, Bacillus* sp., *E. faecalis*, and two fungi species (*Rhodotorula* sp.). The data showed that all isolates displayed antagonistic activity against at least one of the tested pathogens (Table 4), suggesting inhibitory activity was relatively common among extremophiles in salt lakes such as HAALs. Especially, those of *Streptomyces* sp., *Microbacterium* sp., and *Micrococcus* sp. are capable of producing cytotoxic compounds against other organisms.

Undoubtedly, salt lakes are a cradle for polyextremophiles and hold great potential for pharmaceutical applications. The actinobacteria obtained from HAALs are highly UV-resistant and exhibit good antimicrobial activity. The UV-resistance and DNA-regulatory proteins are potentially beneficial to the development of antioxidants and, therefore, should be further investigated.

## 4. Discussion

Based on the different extreme environments discussed in this review, deserts and the deep sea are the most favorable environments for the isolation of bioactive actinobacteria. Compounds with potential applications in medicine have been yielded from these two habitats. One of the reasons is their high abundance in these habitats. Actinobacteria has a dominant diversity and distribution in arid areas [108], and it is the most dominant phylum (72 to 88%) in the Atacama Desert [109]. Similarly, it has been suggested that actinobacteria make up to about 10% of the bacteria colonizing aggregates in the sea, and their antagonistic activity is significant for their survival [110]. Besides, hot springs posed another excellent source for the isolation of bioactive thermophilic actinobacteria based on the literature findings. However, the research on extremophilic actinobacteria’s medical applications from salt lakes was thus far minimal. More research is needed as salt lakes are potentially an excellent source for beneficial bioactive compounds.

For most studies, it is anticipated that future research scope should identify compounds responsible for the observed bioactivities. A typical approach to extract and purify the bioactive compounds from the bacteria would be through bioassay-guided fractionation [111,112,113]. With the chromatography separation techniques, pure compound isolation can be achieved following elucidation of compound structure [114,115].

Additionally, whole-genome sequencing via next-generation sequencing technology (NGS) can also provide means for evaluating the bacteria’s bioactive capability by studying the biosynthetic gene cluster related to the compounds [116,117,118]. In particular, the streptomycetes possessed a prolific potential to synthesize a significant number of valuable secondary metabolites. It has been reported that the genome of the *Streptomyces* spp. can carry more than 20 to 30 biosynthetic gene clusters affiliated with secondary metabolite production [3,119,120,121]. By utilizing the genome sequencing technique and bioinformatics software, the biosynthetic gene clusters of many actinobacteria can thus be identified [122,123,124,125]. A simple illustration is detecting the gene cluster encoding the biosynthesis of ansamycin compounds in the genome sequence of *Streptomyces* sp. LZ35 [126]. The availability of NGS offers accurate results, which pushes forward the sequencing capacity at an affordable price. As more and more actinobacteria are discovered from the aforementioned special niches, discovering new bioactive compounds can also be accomplished through a genomic approach.

## 5. Conclusions

In conclusion, actinobacteria present in extreme environments are great resources that can contribute to microbial drug discovery. Many studies have proven the bioactive potential of these extremophilic actinobacteria. Nevertheless, further in-depth studies are required to explore the bioactive capabilities of these extremophilic actinobacteria. With this, extremophilic actinobacteria represent an alternative rich source of bioactive compounds that can be harvested to develop novel medicines.

## Figures and Tables

**Table 1:** Summary of bioactivity of actinobacterial strains isolated from the Atacama Desert.

Sampling Site	Strain	Extremophilic Properties	Sample Type	Bioactivity	Compound	IC_50_ or MIC	Reference
Salar de Tara of the Atacama Desert, Chile	*Streptomyces* sp. DB634	Polyextremophilic	Desert soil	Anti-inflammatory activity via human recombinant cyclic AMP (cAMP)-specific phosphodiesterase (PDE-4B2) inhibition	Abenquines A and D	IC_50_ Abenquines A: 4.6 ± 0.2 µM; Abenquines D: 4.2 ± 0.3 µM	[69]
Salar de Tara of the Atacama Desert, Chile	*Streptomyces* sp. C34	Polyextremophilic	Desert soil	Antibacterial activity against *E. coli*, *S. aureus* (MRSA and MSSA) Antitumor activity—inhibition of Hsp90	ChaxamycinD Chaxmycins A–D	MIC *E. coli* and *S. aureus*: <1.21 µg/mL IC_50_ N.A. ^a^	[76]
At a high-altitude location (>5000 m) in Atacama Desert	*Lentzea* sp. H45	Polyextremophilic	Desert soil	Inhibition of HIV-integrase	Lentzeosides A–F	IC_50_ Lentzeoside A > 100 µM; Lentzeoside B > 100 µM; Lentzeoside C: 21 µM; Lentzeoside D: 16 µM; Lentzeoside E: 21 µM; Lentzeoside F > 100 µM	[78]
Saudi Arabian desert	*Streptomyces* sp. DA3-7	Thermotolerant (proposed)	Desert soil	Antibacterial activity against: *E. coli*, *S. typhimurium*, *S. aureus*, *P. vulgaris*, *P. aeruginosa*, *E. faecalis*, *K. pneumoniae* Antifungal activity against: *C. albicans* *S. cerevisiae* *C. neoformans*	Pyridine-2,5-diacetamide	MIC *E. coli*: 31.25 μg/mL; *S. typhimurium*, *S. aureus*, *P. vulagris*, *P. aeruginosa*, and *E. faecalis*: 62.5 μg/mL; *K. pneumoniae*: 125 μg/mL; *C. neoformans*: 31.25 μg/mL; *C. albicans* and *S. cerevisiae*: 62.5 μg/mL	[80]

^a^ Not available.

**Table 2:** Bioactivity of actinobacterial strains isolated from the deep-sea environment.

Sampling Site	Actinobacteria	Strain	Extremophilic Properties	Sample Type	Bioactivity	Extract	Compound	IC_50_ or MIC	Reference
Avilés Canyon in Asturias, Spain	*Streptomyces cyaneofuscatus*	M-169 and M-185	Halotolerant, psychrotolerant, and barotolerant	Coral	Antibiotic activity (>2 pathogens); moderate cytotoxic activity against HeLa and HCT 116 cells	Ethyl acetate extract	N.A. ^a^	N.A.	[86]
	*Micromonospora tulbaghiae*	M-194		Coral					
	*Streptomyces carnosus*	M-207		Coral					
	*Streptomyces carnosus*	M-220		Polychaete					
	*Streptomyces sulfureus*	M-231		Decapod					
	*Myceligenerans cantabricum*	M-193		Starfish	Antibiotic activity against *M. luteus* and *Escherichia coli* only; moderate cytotoxic activity against HeLa and HCT 116 cells				
	*Micromonospora aurantiaca*	M-235		Ofiuroid	Antibiotic activity against *M. luteus* and *Streptococcus pneumoniae* only; moderate cytotoxic activity against HeLa and HCT116 cells				
	*Streptomyces cyaneofuscatus*	M-157 and M-190		Coral	Antibiotic activity (>2 pathogens); strong cytotoxic activity (>50%) against HeLa and HCT 116 cells				
	*Streptomyces albidoflavus*	M-179		Polychaete					
	*Streptomyces cyaneofuscatus*	M-192		Actinia					
	*Pseudonocardia carboxydivorans*	M-227		Sea water					
	*Pseudonocardia carboxydivorans*	M-228		Seawater	Antibiotic activity against *M. luteus* only; moderate cytotoxic activity against HeLa				
	*Micromonospora saelicesensis*	M-237		Ofiuroid					
								
	*Streptomyces setonii*	M-178		Sponge	Antibiotic activity against *Neisseria gonorrhoeae* only; strong cytotoxic activity (>50%) against HeLa and HCT 116 cells				
	*Streptomyces halstedii*	M-204		Ofiuroid	Antimicrobial activity against *Clostridium perfringens* and *Candida krusei* only; strong cytotoxic activity (>50%) against HeLa and HCT116 cells				
	*Streptomyces xiamenensis*	M-186		Coral	Strong cytotoxic activity (>50%) against HeLa and HCT 116 cells				
	*Myceligenerans* *cantabricum*	M-201		Coral	Moderate cytotoxic activity against HeLa and HCT 116 cells				
HBOI collection (from Gulf of Mexico, Caribbean Sea, and east coast of the United States)	*Streptomyces* sp.	R818	Halophilic	Sponge	Antifungal activity against *C. albicans*	N.A.	Urauchimycin D	MIC *C. albicans*: 25 µg/mL	[90]
	*Salinispora* sp.	M864	Halophilic	Sponge	Antibacterial activity against *C. difficile*	Ethyl acetate extract	N.A.	*C. difficile*: 0.125 μg/mL	

^a^ Not available.

**Table 3:** Summary of bioactivity of actinobacterial strains isolated from caves.

Sampling Site	Actinobacteria	Strain	Sample Type	Bioactivity	Reference
Moonmilk cave Grotte des Collemboles, Belgium	*Amycolatopsis* sp.	MMun171	Moonmilk	Antibacterial activity against *E. coli*, *P. aeruginosa*, *C. freundii*, *K. pneumoniae*, *B. subtilis*, *S. aureus*, and *M. luteus*	[97]
	*Kocuria rhizophila*	MMun160			
	*Streptomyces* sp.	MMun141 MMun146 MMun156		Strong antibacterial activity, particularly against *B. subtilis*, *S. aureus*, and *M. luteus*	
Shuanghe Karst Cave, Guizhou province, China	*Streptomyces badius*	S142	Bat guano	Antimicrobial activity against *E. coli*, *S. aureus*, *B. cinerea*	[100]
	*Actinoplanes friuliensis*	S761	Rock soil		

**Table 4:** Summary of bioactivity of actinobacterial strains isolated from salt lakes.

Sampling Site	Actinobacteria	Sample Type	Bioactivity	Reference
Laguna Diamante, Antofalla, Laguna Santa Maria, Laguna Socomp, Tolar Grande, and Salina Grande, Argentina	Actinobacterial strains of 11 genera *Streptomyces*, *Micrococcus*, *Microbacterium*, *Nesterenkonia*, *Kocuria*, *Rhodococcus*, *Arthrobacter*, *Micromonospora*, *Blastococcus*, *Brevibacterium*, and *Citricoccus*	Soil, stromatolite, sediment, water, and flamingo feces	Antibacterial activity against *E. coli*, *Bacillus*, *E. faecalis*, *S. aureus*, and *Rhodotorula* sp. (at least 1)	[105]
